# Working conditions, psychological distress and suicidal ideation: cross-sectional survey study of UK junior doctors

**DOI:** 10.1192/bjo.2023.619

**Published:** 2023-12-15

**Authors:** Kevin Rui-Han Teoh, Alice Dunning, Anna Kathryn Taylor, Anya Gopfert, Carolyn A. Chew-Graham, Johanna Spiers, Louis Appleby, Maria Van Hove, Marta Buszewicz, Ruth Riley

**Affiliations:** Department of Organizational Psychology, Birkbeck, University of London, UK; School of Health and Related Research, University of Sheffield, UK; Leeds Institute of Health Sciences, Faculty of Medicine and Health, University of Leeds, UK; Department of Sports Sciences and Public Health, University of Exeter, UK; Department of Sports Sciences and Public Health, Exeter University, UK; School of Health Sciences, University of Surrey, UK; Division of Psychology and Mental Health, School of Medicine, University of Manchester, UK; Department of Health and Community Sciences, University of Exeter, UK; UCL Great Ormand Street Institute of Child Health, University College London, UK

**Keywords:** Depressive disorders, rating scales, risk assessment, suicide, anxiety or fear-related disorders

## Abstract

**Background:**

Evidence attests a link between junior doctors’ working conditions and psychological distress. Despite increasing concerns around suicidality among junior doctors, little is known about its relationship to their working conditions.

**Aims:**

To (a) establish the prevalence of suicidal ideation among junior doctors in the National Health Service; (b) examine the relationships between perceived working conditions and suicidal ideation; and (c) explore whether psychological distress (e.g. symptoms of depression and anxiety) mediates these relationships.

**Method:**

Junior doctors were recruited between March 2020 and January 2021, for a cross-sectional online survey. We used the Health and Safety Executive's Management Standards Tool; Depression, Anxiety and Stress Scale 21; and Paykel Suicidality Scale to assess working conditions, psychological distress and suicidality, respectively.

**Results:**

Of the 424 participants, 50.2% reported suicidal ideation, including 6.1% who had made an attempt on their own life. Participants who identified as LGBTQ+ (odds ratio 2.18, 95% CI 1.15–4.12) or reported depression symptoms (odds ratio 1.10, 95% CI 1.07–1.14) were more likely to report suicidal ideation. No direct relationships were reported between working conditions (i.e. control, support, role clarity, strained relationships, demand and change) and suicidal ideation. However, depression symptoms mediated all six relationships.

**Conclusions:**

This sample of junior doctors reported alarming levels of suicidal ideation. There may be an indirect relationship between working conditions and suicidal ideation via depressive symptoms. Clearer research exploring the experience of suicidality in junior doctors is needed, including those who identify as LGBTQ+. Systematic interventions addressing working environment are needed to support junior doctors’ mental health.

The challenging working conditions and poor mental health of junior doctors in the National Health Service (NHS) are well established.^1–4^ Indeed, the British Medical Association^5^ and General Medical Council^6^ have called for urgent action to improve working conditions to support the mental health and retention of junior doctors. Against this backdrop, the unfortunate cases of junior doctors dying by suicide has raised questions about the role their working conditions may have played in relation to their mental health and suicidality.^7,8^

## Working conditions and suicide

Occupational differences in terms of suicide risk^9^ implicates occupational factors as contributors to suicide. Suicidal ideation refers to having thought about, planned or attempted suicide,^10^ and is an antecedent to dying by suicide.^11^ Growing evidence attests to a relationship between working conditions and suicidal ideation, and to a lesser extent, with death by suicide. In a meta-analysis of 22 studies published over 10 years, job insecurity, demands, low control and lack of colleague and supervisor support were all associated with more cases of suicidal ideation,^12^ although only lack of control and lack of colleague and supervisor support predicted actual suicides. Similarly, a separate review of 12 studies found a positive association between workplace bullying and suicidal ideation.^13^ Although most of the studies in these reviews are cross-sectional, longitudinal studies also corroborate these findings.^14,15^

## Doctors and suicide

Although there is debate on whether doctors are at greater risk of suicide,^9,16,17^ the high levels of psychological distress reported in recent years and certain occupational risk factors are all salient for junior doctors. For example, exposure to traumatic events, making a medical error or being the subject of an investigation are all potential trigger factors for significant distress in junior doctors,^18,19^ and their understanding of human anatomy and access to medical substances provides means and knowledge to facilitate suicide.^20^ Moreover, it has been postulated that medical training encourages competitiveness and perfectionism, which in turn, can increase stigma, reduce help-seeking behaviour and facilitate unhealthy coping styles.^1,7,16,18^

In terms of working conditions and suicidal ideation, doctors from Germany and Norway who reported poor working conditions were 1.92 times more likely to report suicidal ideation.^21^ When focusing on specific aspects of work, fear of litigation, work–life conflict and bullying were all associated with increased suicidal ideation among junior doctors in Australia,^19^ and those working >55 h a week were twice as likely to report suicidal ideation than those working 40–44 h.^22^ Similarly, doctors in Italy and Sweden who reported harassment at work, inadequate resources and stressful situations had an increased likelihood of suicidal ideation.^23^ The increased demands and pressures brought on by the COVID-19 pandemic accentuated many of these aspects, leading to a need for better understanding of the implications of working conditions on the mental health of junior doctors, including suicidality.

## Psychological distress as a mediator between working conditions and suicidality

Despite these findings, reviews and meta-analyses also report substantial heterogeneity in individual studies, and the evidence for some aspects of work, including bullying, job strain and social isolation, to predict suicidality is limited and inconsistent.^12,20^ For example, in a study of 94 000 nurses in the USA, those reporting severe or minimal work stress had twice the number of suicides over the subsequent 14-year period compared with those reporting moderate levels of stress, indicating a U-shaped relationship between work and suicides.^15^ This suggests the need for a more nuanced exploration of the link between working conditions and suicidality, which considers indirect associations and moderators.^11^ It is postulated that that the pathway between working conditions and suicidal ideation travels via the experience of psychological distress, indicating a link between poor working conditions and increased psychological distress^24^ that, in turn, increases the likelihood of suicidal ideation.^25,26^ This is particularly concerning for junior doctors, given the accumulating evidence showing that poor working conditions are associated with more psychological distress.^2,3,27^ Although studies exist that have measured both psychological distress and suicidal ideation among junior doctors,^19,22^ we are aware of only one study where these have been empirically linked, whereby severe depressive symptoms during medical school predicted suicidal planning among first-year doctors in Norway.^14^

## Aims

The increasingly challenging working conditions faced by junior doctors and subsequent concern about their mental health^1,28^ have increased discussion around junior doctor suicides. Therefore, this study aimed to (a) establish the prevalence for suicidal ideation in a sample of junior doctors in the NHS; (b) examine the relationships between junior doctors’ perceived working conditions and suicidal ideation; and (c) explore whether junior doctors’ psychological distress (i.e. symptoms of depression and anxiety) mediates these relationships.

## Method

### Study design and sample

This cross-sectional online survey, administered through Qualtrics(version 2020, Qualtrics, Provo, UT; see https://www.qualtrics.com), ran from March 2020 to January 2021, was part of a wider mixed-methods study focusing on the working experience of junior doctors in the NHS and its implications for their mental health.^1,3,29^ Participants were recruited between March 2020 and January 2021. This wider study was planned before the COVID-19 pandemic, and the survey launched in the initial onset of the pandemic, which meant that pandemic-specific variables were not included. The study was publicised via social media, junior doctor forums and emails circulated via postgraduate medical faculties inviting participants to take part. Three £50 shopping vouchers were offered as part of a prize draw, in recognition of participants’ time.

### Measures

Participants provided sociodemographic information (age, gender, ethnicity, sexuality and tenure – ‘years working as a doctor’) along with measures of their working conditions, depressive and anxiety symptoms, and suicidal ideation.

### Perceived working conditions

We used the UK Health and Safety Executive's (HSE) management standards framework to measure six key aspects of the working environment: work demands, control over the working environment, support in the workplace, strained relationships, role clarity and change. This 35-item scale requires participants to rate how often they experienced each item within the past 6 months, on a five-point Likert scale.

### Mental health

Levels of depression and anxiety symptoms were measured with the corresponding subscales from the Depression, Anxiety and Stress Scale (DASS-21).^30^ The DASS-21 is widely used to assess for the levels of self-perceived depression, anxiety and stress in participants aged 14 years and above. Participants used a four-point Likert scale to rate the extent to which they had experienced each item within the past week. In line with scoring instructions, each seven-item subscale was summed and then multiplied by two to obtain a score (ranging from 14 to 42), with a higher score indicating more severe experiences of anxiety and depression symptoms.

The five-item Paykel Suicidality Scale^10^ was used to assess suicidal ideation. The measure asks participants about experiencing suicidal thoughts and attempts with a yes or no answer. Participants who answered no on all five measures were classed as having ‘no ideation’, those who responded ‘yes’ to either of the first two items of the measure were classed as having a ‘death ideation’. Participants with ‘suicidal ideation’ were those who responded yes to any one of the final three items.

### Ethical considerations

We assert that all procedures contributing to this work comply with the ethical standards of the relevant national and institutional committees on human experimentation and with the Helsinki Declaration of 1975, as revised in 2008. All procedures involving human participants were approved by the University of Birmingham ethics committee and the Health Research Authority (reference number: 19/HRA/6579). Participants were presented with the study information and provided consent via a checkbox before completing the online survey.

### Data analysis

We used SPSS (version 26 for Windows) to analyse descriptive and inferential data. The internal consistency of study measures were analysed with Cronbach's alpha. There was no missing data among the study variables. All of the measures were skewed with kurtosis higher than the 2.0 absolute value, meaning that normality could not be established. As such, we used Spearman's rho correlations between the study variables and chi-squared tests to test against the classified scores.

To examine the direct and indirect relationships between the study variables, we used Hayes PROCESS Model 4 Macro (version 3.5 for Windows; Andrew Hayes, Calgary, Canada; see https://processmacro.org/download.html).^31^ We ran a separate model for each of the six working conditions as the predictor variable, with suicidal ideation as the outcome. Depression and anxiety symptoms were included as mediators in each model. This allowed us to use a calculation of 1000 bias-corrected bootstrapped 95% confidence intervals to test the indirect associations of both mediators. Bootstrapping repeatedly samples the study participants to estimate model parameters and their standard errors, and does not assume that sampling distributions are normal,^32^ making them especially congruent to mediation analyses. Participants’ age, gender, ethnicity, sexuality and tenure were included as control variables.

## Results

We received 424 responses, of which 69.6% identified as female. The sample mean age was 30.70 (s.d. = 4.86) and mean years as a doctor was 4.99 (s.d. = 3.63). Most participants identified as being from a White (71.70%) or Black (15.80%) background (Table 1), with 80.9% identifying as heterosexual. With the exception of the overrepresentation of female participants (69.6% *v*. 48% for the general population), the available demographics are consistent with the medical population.^33^ However, weighting did not change the study results. Chi-squared analysis (*χ*^2^ = 130.96, d.f. = 2; *P* < 0.001) showed that most junior doctors reported suicidal ideation (*N* = 213, 50.2%), followed by no ideation (*N* = 179, 42.2%) and death ideation (*N* = 32, 7.5%). A ‘yes’ response to any of items 3, 4 or 5 in Table 2 were classed as suicidal ideation, whereas participants who only responded ‘yes’ to items 1 and 2 were classed as having ‘death ideation’.

**Table 1:** Sample distribution mapped against suicidal ideation categorisation

Sample characteristic		Suicidal ideation		
	Total *N*	No	Yes	*χ^2^*(d.f.)
Total sample	414 (100%)	211 (49.8%)	213 (50.2%)	0.01 (1)
Gender				3.16 (3)
Male	115 (28%)	58 (50.4%)	57 (49.6%)	
Female	286 (69.6%)	144 (50.3%)	142 (49.7%)	
Prefer to self-describe	3 (0.7%)	–	3 (100%)	
Prefer not to say	7 (1.7%)	4 (57.1%)	3 (42.9%)	
Ethnicity				2.97 (4)
White	303 (71.5%)	144 (68.2%)	159 (52.5%)	
Asian	67 (15.8%)	36 (53.7%)	31 (46.3%)	
Black	7 (1.7%)	5 (71.4%)	2 (28.6%)	
Mixed	26 (6.1%)	14 (53.8%)	12 (46.2%)	
Other	21 (5%)	12 (57.1%)	9 (42.9%)	
Sexuality				8.15 (2)*
Heterosexual	339 (80.9%)	180 (53.1%)	159 (46.9%)	
Lesbian, gay or bisexual	65 (15.5%)	22 (33.8%)	43 (66.2%)	
Prefer not to say	15 (3.6%)	7 (46.7%)	8 (53.3%)	

* *P* < 0.05.

**Table 2:** Distribution of participant responses on suicidality aspects

Ideation	Suicidality item (Have you ever … )	Yes, *n* (%)	No, *n* (%)	*χ^2^*(*d.f.*)
Death	Felt that life was not worth living?	210 (46.1%)	214 (46.9%)	0.04 (1)
Death	Wished you were dead?	168 (36.8%)	256 (56.1%)	18.26 (1)***
Suicidal	Thought of taking your own life, even if you would not really do it?	211 (46.3%)	213 (46.7%)	0.01 (1)
Suicidal	Reached the point where you seriously considered taking your life, or perhaps made plans how you would go about doing it?	99 (21.7%)	325 (71.3%)	120.46 (1)***
Suicidal	Made an attempt to take your own life?	28 (6.1%)	396 (86.8%)	319.40 (1)***

*** *P* < 0.01.

Table 1 presents full demographic details, as well as the demographic breakdown of responses to suicidal ideation and non-suicidal ideation (i.e. no ideation and death ideation together). No demographic differences on suicidal ideation were reported based on gender and ethnicity, although a higher proportion of participants who identified as lesbian, gay or bisexual reported suicidal ideation (66.2%).

Table 2 provides an overview of the proportion of participants who reported five different aspects of suicidality. Although most participants did not report individual aspects of suicidality, the results shows that one out of three (36.8%) participants had wished they were dead, one out of five had considered taking their own life (21.7%) and one out of 20 (6.1%) disclosed making an attempt to take their own life. Although overall suicidal ideation was 50.2%, Table 2 shows that this was largely because of the response on one item, where 46.3% reported thinking of taking their own life even if they would not really do it.

### Correlates of suicidal ideation

The internal reliabilities for the study variables are presented in Table 3. Based on univariate logistic regression analysis, several variables were found to change the odds ratio of suicidal ideation. Multivariate analysis show that participants who identified as lesbian, gay or bisexual were twice as likely to report suicidal ideation (odds ratio 2.18, 95% CI 1.15–4.12; *P* < 0.05) compared with those who did not. More severe depression symptoms also increased the odds ratio of suicidal ideation (odds ratio 1.10, 95% CI 1.07–1.14; *P* < 0.001) (Table 3). None of the perceived working conditions were significant correlates of suicidal ideation within the multivariate analysis.

**Table 3:** Reliability coefficients for study variables and univariate and multivariate correlates of suicidal ideation

	Internal reliability (Cronbach's *α*)	Univariate odds ratio (95% CI)	Multivariate odds ratio (95% CI)
Gender (1 = male)	–	0.99 (0.65–1.54)	0.92 (0.54–1.56)
Age	–	1.04 (0.99–1.08)	1.05 (0.98–1.11)
Years as a doctor	–	1.03 (0.78–1.10)	0.97 (0.88–1.07)
Ethnicity (1 = White)	–	1.34 (0.87–2.05)	1.51 (0.88–2.60)
Sexuality (1 = LGB)	–	2.17*** (1.25–23.78)	2.18* (1.15–4.12)
Demands	0.86	1.64*** (1.23–2.20)	1.25 (0.78–1.99)
Control	0.85	0.78 (0.60–1.02)	1.12 (0.69–1.82)
Support	0.91	0.73** (0.57–0.93)	1.42 (0.87–2.32)
Strained relationships	0.84	1.56*** (1.21–2.02)	0.87 (0.57–1.33)
Change	0.80	0.71 (0.55–0.90)	0.72 (0.49–1.10)
Role clarity	0.78	0.79 (0.57–1.10)	1.63 (0.98–2.72)
Depression	0.92	1.09*** (1.06–1.11)	1.10*** (1.07–1.14)
Anxiety	0.79	1.07*** (1.04–1.10)	1.03 (0.99–1.07)

LGB, lesbian, gay or bisexual.

*P < 0.05, **P < 0.01, ***P < 0.01.

### Indirect associations between perceived working conditions and suicidal ideation

All six perceived working conditions were observed to predict severity of depression and anxiety symptoms. More specifically, demands and strained relationships had a positive association with both outcome measures (Table 4), whereas control, support, role clarity and change negatively predicted depression and anxiety symptoms. The strongest predictors of depression symptoms were strained relationships (*b* = 7.44, 95% CI 6.25–8.62), (lack of) support (*b* = −7.00, 95% CI −8.21 to −5.79) and (lack of) role clarity (*b* = −6.84, 95% CI −5.17 to −2.45). For anxiety, the strongest predictors were strained relationships (*b* = 4.63, 95% CI 3.68–5.58), demands (*b* = 5.29, 95% CI 3.37–5.66) and (lack of) role clarity (*b* = −3.81, 95% CI −5.17 to −2.45). Depression symptoms predicted suicidal ideation across all six of the tested models (between *b* = 0.08 and *b* = 0.09). In contrast, anxiety symptoms did not predict suicidal ideation, with effect sizes of between *b* = 0.01 and *b* = 0.02 across the six models.

**Table 4:** Estimated coefficients for indirect effects between perceived working conditions and suicidal ideation

		Mediator: depression			Mediator: anxiety			
Predictor	Effect^a^ of predictor on suicidal ideation	Effect^b^ of predictor on depression	Effect^a^ of depression on suicidal ideation	Indirect effect^b^ of predictor on suicidal ideation	Effect^b^ of predictor on anxiety	Effect^a^ of anxiety on suicidal ideation	Indirect effect^b^ of predictor on suicidal ideation	Total effects^b^ on suicidal ideation
Demands	−0.02 (−0.36 to 0.33)	6.67 (5.18−8.15)*	0.08 (0.05−0.10)*	0.51 (0.33−0.77)*	4.51 (3.37−5.66)*	0.01 (−0.02 to 0.05)	0.06 (−0.10 to 0.22)	0.57 (0.40−0.80)*
Control	0.22 (−0.10 to 0.54)	−5.51 (−6.96 to −4.07)*	0.08 (0.06−0.11)*	−0.45 (−0.69 to −0.27)*	−2.88 (−4.02 to −1.75)*	0.01 (−0.02 to 0.05)	−0.04 (−0.17 to 0.05)	−0.49 (−0.74 to −0.30)*
Support	0.30 (−0.01 to 0.62)	−7.00 (−8.21 to −5.79)*	0.09 (0.06−0.12)*	−0.61 (−0.87 to −0.41)*	−3.47 (−4.47 to −2.48)*	0.01 (−0.02 to 0.05)	−0.05 (−0.19 to 0.05)	−0.66 (−0.95 to −0.47)*
Strained relationships	−0.18 (−0.51 to 0.14)	7.44 (6.25−8.62)*	0.08 (0.05−0.11)*	0.61 (0.40−0.88)*	4.63 (3.68−5.58)*	0.02 (−0.02 to 0.05)	0.07 (−0.08 to 0.25)	0.69 (0.48−0.96)*
Role clarity	0.38 (−0.02 to 0.78)	−6.84 (−5.17 to −2.45)*	0.08 (0.06−0.11)*	−0.57 (−0.84 to −0.35)*	−3.81 (−5.17 to −2.45)*	0.02 (−0.02 to 0.05)	−0.06 (0.21−0.25)	−0.63 (−0.93 to −0.42)*
Change	0.01 (−0.29 to 0.26)	−4.63 (−5.93 to −3.34)*	0.08 (0.05−0.10)*	−0.38 (−0.58 to −0.23)*	−1.79 (−2.81 to −0.77)*	0.01 (−0.02 to 0.04)	−0.02 (−0.11 to 0.03)	−0.40 (−0.60 to −0.26)*

Gender, age, experience, ethnicity and sexuality were included as covariates.

a. Log-odds metric coefficients. Parentheses represent 95% confidence intervals.

b. Unstandardised coefficients.

* *P* < 0.05.

Congruent with the multivariate logistic regression (Table 3), none of the six perceived working conditions predicted suicidal ideation. Table 4 shows instead an indirect association via depression symptoms, where in all six models, depression symptoms mediated the relationship between perceived working conditions and suicidal ideation. More specifically, better perceived working conditions were associated with lower levels of depression, which, in turn, was associated with reduced likelihood of reporting suicidal ideation (between *b* = −0.38 and *b* = −0.61). The converse was observed for demands (*b* = 0.51) and strained relationships (*b* = 0.61), which were positively correlated with depression, and, in turn, were also positively correlated with suicidal ideation. Anxiety symptoms did not mediate any of the six relationships tested.

## Discussion

Our findings indicate that half of junior doctors surveyed during the COVID-19 pandemic reported suicidal ideation, with 21.7% having considered taking their own life and 6.1% reporting an attempt to take their own life, which are very troubling findings. Participants who reported more severe depression symptoms or who identified as lesbian, gay or bisexual were more vulnerable to suicidal ideation. The study did not find any direct relationships between the six working conditions examined (control, support, role clarity, strained relationships, demand and change) and suicidal ideation. However, indirect relationships were observed where these six working conditions were associated with increased severity of depression symptoms, which, in turn, increased the likelihood of suicidal ideation. These findings build on the literature to further our understanding of a possible pathway between the working environment and suicidal ideation, and further emphasise the need to address the factors that contribute to poor mental health in junior doctors.

### The relationship between working conditions and suicidal ideation

We did not find a direct relationship between working conditions and suicidal ideation. This may be a function of the use of HSE Management Standards Tool to measure the six aspects of the working environment. This tool was designed for workplaces in general, and so may lack the specificity required to capture the working environment of junior doctors. For example, other work factors or even trigger events identified in the literature (e.g. exposure to trauma, moral injury, bullying, work–life conflict)^16,19,23^ may have yielded direct relationships with suicidal ideation, especially as there is evidence that different doctor seniority levels (e.g. resident, specialist) have different antecedents to suicidal ideation.^34^

The complexity of a potential relationship between working conditions and suicidal ideation is evident, in that depression, but not anxiety symptoms, was a mediator for all six working conditions examined. The influence of working conditions on depression in doctors is well established,^3,35^ with the wider research literature indicating a link between depression and suicidal ideation.^20^ This may be because depression accentuates feelings of defeat and entrapment that contribute to the development of suicidal ideation. Moreover, depression can impair problem-solving and appropriate coping, as well as increase rumination,^36^ all of which potentially raise feelings of entrapment. Similarly, depression also undermines feelings of belonging, goal-setting and social support, which also increases the likelihood of suicidal ideation.^11^

These findings emphasise the complexity of this relationship, with the need for models that capture and explain the factors within it. In particular, suicidal ideation is a known problematic outcome to measure, and is more strongly associated with distress than actual suicidal behaviour.^37^ Although our analyses show low likelihood of multiple collinearities between the measures of depression symptoms, anxiety symptoms and suicidal ideation, all three constructs are indicators of psychological distress. Therefore, any conclusions drawn from this study with regards to death by suicide and suicide risk must be cautious.

### Individual differences on suicidal ideation

This was the first study to show an association between sexuality and suicidality among junior doctors and is congruent with the extant literature indicating higher rates of suicidal ideation and attempts among LGBTQ+ groups compared with the general population.^38,39^ These findings echo those of related studies showing higher levels of burnout among ophthalmologists^40^ and anaesthesiologists^39^ who identify as LGBTQ+ than those who do not. The underlying factors explaining this are complex, and there is a clear need to better understand the experience of LGBTQ+ junior doctors, including the role of workplace factors and their interaction with non-work factors as antecedents to poor mental health. Harassment, fear of discrimination from colleagues and patients, and the psychological stress of disclosure have all been identified as possible contributing factors.^39^ There is a role for organisations and stakeholders to develop interventions and policies to promote supportive workplaces that address both workplace inequality and discrimination in general, as well as to promote and advocate for LGBTQ+ inclusion.^38,39^ This includes efforts to create a psychologically safe space and address stigma, including addressing systematic prejudice within the medical curriculum and delivery,^41^ and supporting staff mental health and well-being in a meaningful way.

We found no gender differences in suicidal ideation. The measure of suicidal ideation used here consisted of items about thoughts and planning of suicide, as well as about suicide attempts. This differs to the existing literature on gender differences, which focuses on actual suicide rates^9,42^ and suggests the need to differentiate between ideation and actual deaths by suicide, with possible moderators (e.g. access to means, impulsivity, prior exposure to suicide) that could intervene between ideation and actual behaviour warranting further exploration.^11^

### Study strengths and limitations

A key strength of this study is the use of measures with robust psychometric properties. However, the cross-sectional study design used means that we cannot presume causality. Nevertheless, it is worth noting that although longitudinal research reports a link between working conditions and future levels of suicidal ideation, suicidal ideation at the start of the study did not predict future perceptions of the working environment.^13^ The use of convenience sampling methods, and the inability to calculate a response rate, means that generalising the results from this study should be approached with caution. We also note that surveys on mental health may be overrepresented, in that they attract participants who are more keen to share their related experiences; however, it could also be underrepresented, as junior doctors who are struggling with mental health are more likely to exit the workforce or not have the time to take part.^2^ Given the different time windows for the different measures, there is the potential for confounding between the constructs used, where the DASS-21 covered a more recent (1-week) period but the Paykel Suicidality Scale covered the previous year. The longer time period for suicidality therefore covers more recent (active) and older (passive) ideation, which may differentially relate to the more recent measure of depression and anxiety symptoms. Future research may want to consider self-reported of measures that cover a longer period of time (e.g. the State-Trait Anxiety Inventory).

Finally, we recognise that the bulk of data collection occurred during the first wave of the COVID-19 pandemic, when working conditions were not only challenging, but when the pandemic itself was a potentially significant confounder in participants’ mental health and their experience of suicidal ideation. As this study was conceived before the pandemic, we were unable to control for specific aspects of working through COVID-19, such as the extent to which the individuals were in contact with patients with COVID-19 or had access to adequate personal protective equipment. Therefore, caution is again needed before generalising beyond this context.

### Implications

Although early identification for junior doctors in recognising distress and signposting to support services is imperative, more work is also needed to address stigma around psychological distress among healthcare workers and to create psychologically safe spaces for doctors to seek help.^8,16^ Similarly, better identification of risk and trigger effects would allow for better preparation for staff to respond to such difficulties, and for appropriate debriefing and follow-up.

While the contributing factors toward suicidality among junior doctors are complex and multifaceted, the indirect association of working conditions provides evidence that the impact of working environments should be considered in the aetiology of suicides. Crucially, given the challenges around operationalising links between suicidality and death by suicide, extreme care needs to be taken when drawing conclusions from research in this area. Moving forward, there is a need for more representative participants and designs that better tease apart different measures of psychiatric distress.

Equally, the findings further reinforce the need to create healthier working environments, as these are not only associated with financial costs and patient care outcomes,^43^ but also have implications for the experience of psychological distress in junior doctors, and their possible levels of suicidal ideation. This necessitates senior leaders and stakeholders at a local and national level taking responsibility for providing the required resources for a healthy working environment, including funding, staffing and support services.

In conclusion, the findings in this study show that a sample of junior doctors surveyed during the COVID-19 pandemic reported concerning levels of suicidal ideation, including that one out of five had considered taking their own life and one out of 20 disclosed making an attempt to take their own life. Participants who identified as lesbian, gay or bisexual were particularly vulnerable. No direct relationships were reported between the working environment factors examined and suicidal ideation. Indirect associations were observed, where working conditions were associated with severity of depression symptoms, which, in turn, was associated with suicidal ideation. However, we must be cautious about generalising these findings to the wider junior doctor population, given the sample size and the study design. Although this study was situated during the COVID-19 pandemic, concerns have been raised that working conditions have not improved since.^4^ All of this emphasises the need for urgent action for more systematic interventions to manage and support the mental health of junior doctors.

## Data Availability

The data that support the findings of this study can be available on request from the corresponding author, K.R.-H.T. The data are not publicly available due to information that could compromise the privacy of research participants and the absence of ethical consent to share data.
